# Organizational Health Literacy and Health Among New York State Medicaid Members

**DOI:** 10.3928/24748307-20230822-01

**Published:** 2023-07

**Authors:** Thomas W. Robertson, Jennifer A. Manganello, Meng Wu, Lauren S. Miller, Recai M. Yucel, Anne M. Schettine

## Abstract

**Background:**

The definition of health literacy has recently expanded beyond the idea of individual skills to include the system and environment the individual interacts with to receive care, known as organizational health literacy (OHL). However, neither the prevalence of OHL nor the impact of OHL on individuals' perceptions of their health and healthcare have been examined in New York's Medicaid managed care population.

**Objective:**

This study aimed to estimate the prevalence of organizational health literacy in the New York State (NYS) Medicaid Managed Care (MMC) program.

**Methods:**

A brief measure to assess organizational health literacy was developed from responses to two questions in the 2018 NYS Consumer Assessment of Healthcare Providers and Systems (CAHPS) survey. Generalized Estimating Equation models were developed to analyze the association between organizational health literacy and three aspects of perceptions of health and health care, controlling for demographic differences and clustering effects from health insurance plans. Missing data were handled using multiple imputation.

**Key Results:**

Among 3,598 members included in the study, 20% of the MMC members reported inadequate organizational health literacy. These members were more likely to be older, less educated, from racial and ethnic minority groups, and less fluent with English. They are more likely to have poorer self-reported health (odds ratio [OR] 1.49), lower perceived access to health care (OR 6.97), and lower satisfaction with their health care (OR 6.49) than members who did not report inadequate organizational health literacy.

**Conclusions:**

Our results suggest that a proportion of the NYS MMC population faces inadequate organizational health literacy, which can present a barrier to health care access and result in patients having a significantly poorer health care experience. Using an existing data source that is part of existing data collection allows for routine assessment of organizational health literacy, which can help inform health plans about areas for potential improvement. [***HLRP: Health Literacy Research and Practice*. 2023;7(3):e154–e164.**]

## Plain Language Summary

Our study looked at the impact of organizational health literacy on the perceptions of health and health care of NYS Medicaid Managed Care population. We used existing CAHPS questions to assess organizational health literacy and found that lower organizational health literacy led to worse perceptions of health and health care. This article illustrates an opportunity to demonstrate how organizational health literacy can be measured with annual CAHPS collections.

Health literacy has traditionally been defined as “the capacity to obtain, process, and understand basic health information and services needed to make appropriate health decisions” (U.S. Department of Health and Human Services, Office of Disease Prevention and Health Promotion, 2010). These skills include interpreting documents, reading and writing prose (print literacy), using quantitative information (numeracy), and speaking and listening effectively (oral health literacy; Berkman, Sheridan, Donahue, Halpern, Viera, et al., 2011). In the only United States survey of health literacy conducted in 2003 with 19,000 adults, results showed that low health literacy is a pervasive problem in the U.S., such that 14% of adults had a below basic level of health literacy, and an additional 22% of adults had a basic level of health literacy.

However, in recent years, the idea of health literacy has expanded from a focus only on individual skills to include the system and environment that people interact with (Sørensen et al., 2021). The expansion of the concept of organizational health literacy (OHL) was further developed with a 2012 publication titled Ten Attributes of Health Literate Health Care Organizations (Brach et al., 2012). Since that time, research and practice has expanded on this idea of OHL, defined as “the way in which services, organizations and systems make health information and resources available and accessible to people according to health literacy strengths and limitations.”

There are different ways of defining and measuring OHL (Bremer et al., 2021; Hayran & Ege, 2023). Similar to the assessments of individual health literacy, measures of OHL are varied and there is no one commonly utilized tool. Each tool also measures different aspects of OHL. One of the first attempts to assess the health literacy of an organization was presented in the Health Literacy Environment Activity Packet (Rudd, n.d.). This includes activities such as calling the phone number, visiting the website, and walking through the facility. Other measures have been created since then. One is called the Health Literate Healthcare Organization 10-Item Questionnaire, which assesses the 10 attributes presented in the 2012 report (Kowalski et al., 2015). Another is designed to assess health literacy sensitive communication (Ernstmann et al., 2017; Lubasch et al., 2021). This consists of nine self-report items administered to patients, including questions such as “People spoke slowly and clearly to me.” The Organizational Health Literacy Responsiveness self-assessment tool was designed to assess multiple aspects of organizations including leadership, access to services, and community engagement (Trezona et al., 2018). There is also the Organizational Health Literacy of Hospitals tool (International Working Group Health Promoting Hospitals and Health Literate Healthcare, 2019).

Another tool that has been used is the Consumer Assessment of Healthcare Providers and Systems (CAHPS) Item Sets for Addressing Health Literacy. There are 55 items in the health literacy set for health plans to capture the patients' perspective on how well health information is communicated to them by health care professionals. They are intended to serve as a measure of whether health care professionals have succeeded in reducing the health literacy demands they place on patients (Weidmer et al., 2012), which is a key component of organizational health literacy.

## Impact of Health Literacy

Health literacy is often associated with poor health outcomes (Berkman, Sheridan, Donahue, Halpern, & Crotty, 2011; Taylor et al., 2018). A key area impacted by health literacy is how patients experience the health care system and patient perceptions of health. We focused on three aspects: perceived access to health care, satisfaction with the health care received, and individuals' self-reported health.

### Individual Characteristics

When assessing OHL, one might assume that patient characteristics can influence results for measures using patient self-report items. However, at least one study found that not to be the case (Lubasch et al., 2021). More research is needed to better understand how patient factors play a role in perceptions of health literacy practices.

### Perceived Access to Health Care

Individuals with lower levels of health literacy report less access to health care, even after controlling for patients' insurance status and demographic variables (Levy & Janke, 2016). Perceived access to health care is important for preventing unnecessary care. For example, communities with higher perceived access to health care tend to have lower rates of unneeded hospitalizations (Bindman et al., 1995). OHL also contributes to access to health services (Palumbo, 2021).

### Satisfaction with Health Care

A patient's ability to understand health information can significantly impact their perception of the quality of care they receive and their satisfaction with that care. Individuals with low health literacy typically report lower levels of satisfaction with the health care that they receive (Altin & Stock, 2015, 2016; Aoki & Inoue, 2017; Örsal et al., 2019; Roh et al., 2016). Research also shows that Medicare supplement enrollees with inadequate literacy were more dissatisfied with their overall health care and their physicians (MacLeod et al., 2017). Likewise, OHL has been associated with patient satisfaction and views on care quality as well (Hayran & Özer, 2018; Palumbo, 2021).

### Self-Reports of Health

Low health literacy has consistently been found to be linked to poor self-reported health (Aaby et al., 2017; Chang, 2011; Furuya et al., 2015; Lundetræ & Gabrielsen, 2016; Ownby et al., 2012). As an example, this relationship has been found in the National Assessment of Adult Literacy survey which consists of a large sample representative of adults older than age 40 years in the U.S. (Ownby et al., 2012). The link between low health literacy and poor self-reported health has also been found in countries outside of the United States (Aaby et al., 2017; Chang, 2011; Furuya et al., 2015; Lundetræ & Gabrielsen, 2016). However, it is unclear what role OHL plays on health status self-report.

## Purpose of the Study

The Centers for Medicare and Medicaid Services has recognized the role that low health literacy plays in creating health disparities and has advocated for states to conduct research examining factors, including health literacy, that impact health disparities within the Medicare and Medicaid populations (Centers for Medicare & Medicaid Services, 2016; Chang, 2011). The use of CAHPS with health plans is required for New York State (NYS) reporting requirements and national accreditation programs, providing a rich opportunity to collect OHL information on a large scale.

Yet, resources and time are limited to enable administration of full CAHPS health literacy items. States typically do not administer both regular CAHPS and the CAHPS Health Literacy Item Set due to the cost involved with additional questions and efforts to minimize the burden on respondents. At the same time, there has been an ongoing decrease in responses to CAHPS questions (Tesler & Sorra, 2017). Thus, it is imperative to identify easy ways for large organizations including state health departments to capture even a small look at organizational efforts to enhance health literacy.

To that end, this study sought to develop a brief assessment for OHL using one item from routinely administered CAHPS in NYS and one item from CAHPS Health Literacy Item Set. Second, the study sought to assess the association between OHL and perceived access to care, satisfaction with care and self-reported health. Given the relatively low response rate for CAHPS items and its downward trend (Tesler & Sorra, 2017), we incorporated both non-response bias adjustment and multiple imputation techniques in our analyses to address missing data issues with the purpose of improving statistical power and providing unbiased estimates (Rubin, 1987).

## Methods

### Study Population

The 2018 CAHPS adult survey was available in English and Spanish and administered to a random sample of 1,500 adult members from each of the 15 MMC plans in New York, Surveys were sent to 22,500 members following a combined mail and phone methodology (three postal mailings, followed by phone follow up of nonresponders) during the period from October 3, 2017 to January 7, 2018. A total of 5,048 responses were received, resulting in a 23.8% response rate. Respondents were excluded if they skipped the health literacy questions due to not seeing a doctor or having a medical test performed. Our final sample contained 3,598 members.

## Measures

### Organizational Health Literacy

The regular CAHPS and CAHPS Health Literacy Item Set contain validated survey items for organizations to monitor their OHL status from the patient perspective. In this study, we attempted to use two items to derive a composite measure as a brief screener for OHL, targeting oral communication and understanding of written information. One question is from the regular CAHPS survey (“In the last 6 months, how often did your personal doctor explain things in a way that was easy to understand?”) and the other one is from the CAHPS Health Literacy Item Set (“In the last 6 months, how often were the results of your blood test, X-ray or other test easy to understand?”; New York State Department of Health, 2018a). Both questions are 4-point Likert-type items ranging from *Never* to *Always*. Due to limited space under the established contract for the regular CAHPS, only one additional question from the health literacy set can be added to the survey. The selected item allowed us to evaluate the impact of challenges in understanding of health information. Organizations were categorized as having inadequate health literacy if patients responded *Never* or *Sometimes *to at least 1 of the 2 questions. The classification was imputed if a participate did not answer both questions. This composite measure was used as an indicator of OHL as reported by patients.

### Perceptions of Health

Three outcome variables were derived from the survey. Low perceived access to care was identified using a 4-point Likert-type item when members responded *Never* or *Sometimes *to the question, “In the last 6 months, how often was it easy to get the care, tests, or treatment you needed?” Poor self-reported health was identified on a five-point Likert item when members responded *Fair* or *Poor *to the question, “In general, how would you rate your overall health?” Dissatisfaction with health care was identified as a score of 6 or less on a 10-point scale to the question: “Using any number from 0 to 10, where 0 is the worst health care possible and 10 is the best health care possible, what number would you use to rate all your health care in the last 6 months?”

### Covariates

Several demographic and health characteristic variables were included in the model to control for potentially confounding effect. From the CAHPS survey, age, gender, education, race and ethnicity, spoken English ability, smoking status, chronic physical health condition, mental health condition, or other health condition lasting more than 3 months. A small proportion of participants did not respond to some of these questions and missing values were coded as being in the unknown category.

### Statistical Analysis

We conducted bivariate analyses to examine whether there were statistically significant differences in demographic and health characteristics when comparing responses about OHL.

To account for correlated observational units within each sampled health plan, a Generalized Estimating Equation model was fit for each perception of health outcome with health literacy as the exposure of interest and a set of covariates as described above. To address unit non-response bias, a covariate adjustment propensity score method was applied (D'Agostino, 1998). Propensity scores were calculated using a logistic regression based on race, age, gender, language spoken, and residence. For the respondents included in the study, we found 14% had item non-response with either the outcome or exposure variables, this was handled using multiple imputation (Robitzsch et al., 2018; Rubin, 1987; Schafer & Graham, 2002; van Buuren, 2018; van Buuren & Groothuis-Oudshoorn, 2011).

Finally, we examined how OHL varied across MMC plans. A logistic regression was employed to estimate plan's average OHL, adjusting for member's demographics. A proportional *z* test was used to identify plans with significant difference compared to the statewide average. We grouped plans into three groups based on the comparison (lower, higher, no difference), and summarized their demographics distribution.

## Results

The demographics of the study cohort are summarized in **Table 1**. The respondents were racially diverse with White (36%), Latino/a/e and Hispanic (25%), Asian, Native Hawaiian/Pacific Islander, and American Indian/Alaska Native (20%), African American/Black (16%) and unknown race (4%). More female respondents (60%) than male respondents (37%) participated, and a small percent (3%) did not report their sex. Ages ranged from 18 to older than 74 years. Different health characteristics were also present (**Table 2**); 23% reported smoking and many participants had a self-reported chronic (62%), mental health (27%), and/or other health condition (32%).

**Table 1:**
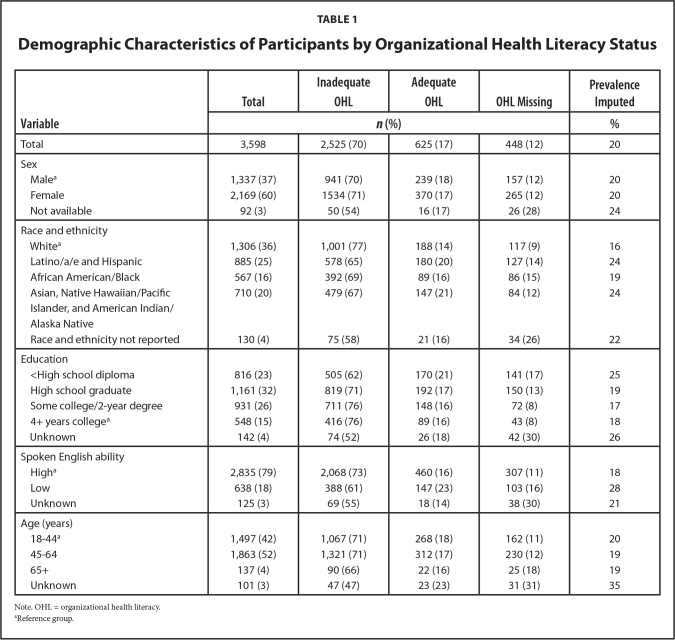
Demographic Characteristics of Participants by Organizational Health Literacy Status

**Variable**	**Total**	**Inadequate OHL**	**Adequate OHL**	**OHL Missing**	**Prevalence Imputed**

	***n* (%)**				**%**

Total	3,598	2,525 (70)	625 (17)	448 (12)	20

Sex					
Male^a^	1,337 (37)	941 (70)	239 (18)	157 (12)	20
Female	2,169 (60)	1534 (71)	370 (17)	265 (12)	20
Not available	92 (3)	50 (54)	16 (17)	26 (28)	24

Race and ethnicity					
White^a^	1,306 (36)	1,001 (77)	188 (14)	117 (9)	16
Latino/a/e and Hispanic	885 (25)	578 (65)	180 (20)	127 (14)	24
African American/Black	567 (16)	392 (69)	89 (16)	86 (15)	19
Asian, Native Hawaiian/Pacific	710 (20)	479 (67)	147 (21)	84 (12)	24
Islander, and American Indian/Alaska Native					
Race and ethnicity not reported	130 (4)	75 (58)	21 (16)	34 (26)	22

Education					
<High school diploma	816 (23)	505 (62)	170 (21)	141 (17)	25
High school graduate	1,161 (32)	819 (71)	192 (17)	150 (13)	19
Some college/2-year degree	931 (26)	711 (76)	148 (16)	72 (8)	17
4+ years college^a^	548 (15)	416 (76)	89 (16)	43 (8)	18
Unknown	142 (4)	74 (52)	26 (18)	42 (30)	26

Spoken English ability					
High^a^	2,835 (79)	2,068 (73)	460 (16)	307 (11)	18
Low	638 (18)	388 (61)	147 (23)	103 (16)	28
Unknown	125 (3)	69 (55)	18 (14)	38 (30)	21

Age (years)					
18–44^a^	1,497 (42)	1,067 (71)	268 (18)	162 (11)	20
45–64	1,863 (52)	1,321 (71)	312 (17)	230 (12)	19
65+	137 (4)	90 (66)	22 (16)	25 (18)	19
Unknown	101 (3)	47 (47)	23 (23)	31 (31)	35

Note. OHL = organizational health literacy.

a Reference group.

**Table 2:**
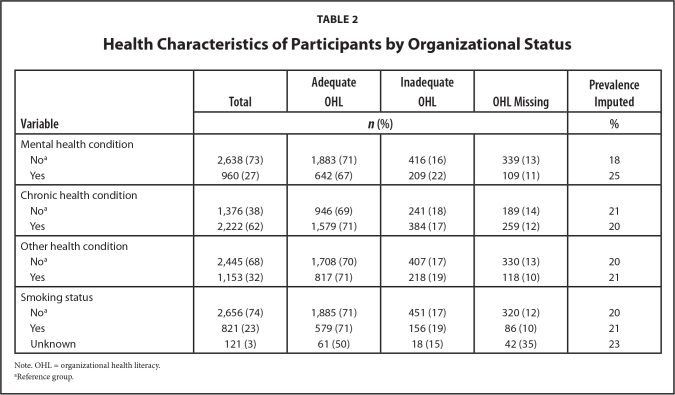
Health Characteristics of Participants by Organizational Status

**Variable**	**Total**	**Adequate OHL**	**Inadequate OHL**	**OHL Missing**	**Prevalence Imputed**
	** **					
	***n* (%)**				**%**

Mental health condition					
No^a^	2,638 (73)	1,883 (71)	416 (16)	339 (13)	18
Yes	960 (27)	642 (67)	209 (22)	109 (11)	25

Chronic health condition					
No^a^	1,376 (38)	946 (69)	241 (18)	189 (14)	21
Yes	2,222 (62)	1,579 (71)	384 (17)	259 (12)	20

Other health condition					
No^a^	2,445 (68)	1,708 (70)	407 (17)	330 (13)	20
Yes	1,153 (32)	817 (71)	218 (19)	118 (10)	21

Smoking status					
No^a^	2,656 (74)	1,885 (71)	451 (17)	320 (12)	20
Yes	821 (23)	579 (71)	156 (19)	86 (10)	21
Unknown	121 (3)	61 (50)	18 (15)	42 (35)	23

Note. OHL = organizational health literacy.

a Reference group.

The overall estimated prevalence of inadequate OHL was 20%, with the highest among Latino/a/e and Hispanic participants (24%), those with less than high school education (25%), low ability of spoken English (28%), and participants with mental health condition(s) (25%). Compared to other groups, these groups had significantly higher rates (*p* < .001).

Participants who reported inadequate OHL were 6.97 times more likely to experience lower access to care, 6.49 times more likely to be dissatisfied with health care, and 1.49 times more likely to report poor self-reported health (**Table 3**). For the covariates, the results show that Latino/a/e and Hispanic members and members who had a mental health condition were more likely to report lower access to health care, with an odds ratio (OR) of 1.44 and 1.33 respectively. In addition, members who did not have a high school diploma (OR 2.11), members with lower English language ability (*OR* = 1.76), Latino/a/e and Hispanic members (*OR* = 1.46), members between ages 45 and 64 years (*OR* = 1.29), members with a chronic health (*OR* = 3.30), mental health (*OR* = 2.23) or other health (OR=3.30) conditions, and members who smoked (*OR* = 1.38) were more likely to report poor health compared to their reference group.

**Table 3:**
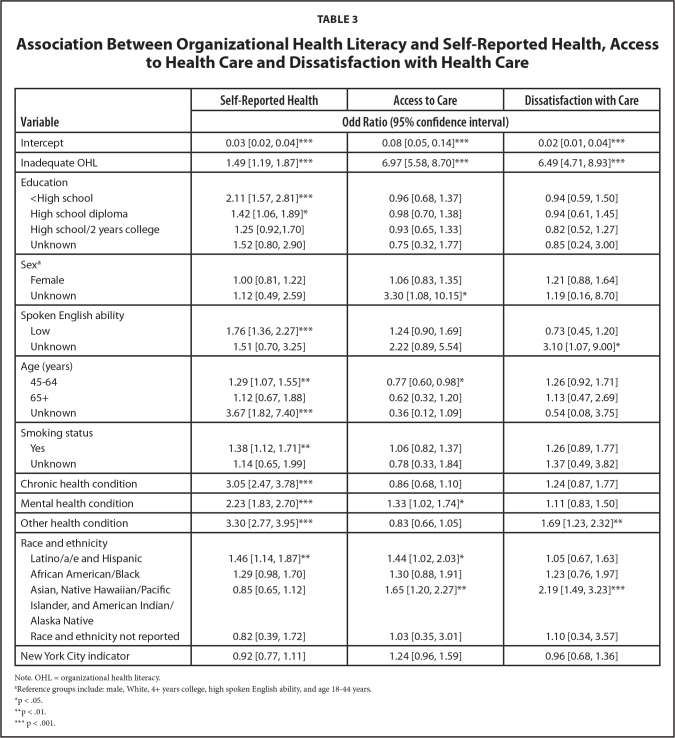
Association Between Organizational Health Literacy and Self-Reported Health, Access to Health Care and Dissatisfaction with Health Care

**Variable**	**Self-Reported Health**	**Access to Care**	**Dissatisfaction with Care**

	**Odd Ratio (95% confidence interval)**		

Intercept	0.03 [0.02, 0.04]^***^	0.08 [0.05, 0.14]^***^	0.02 [0.01, 0.04]^***^

Inadequate OHL	1.49 [1.19, 1.87]^***^	6.97 [5.58, 8.70]^***^	6.49 [4.71, 8.93]^***^

Education			
<High school	2.11 [1.57, 2.81]^***^	0.96 [0.68, 1.37]	0.94 [0.59, 1.50]
High school diploma	1.42 [1.06, 1.89]^*^	0.98 [0.70, 1.38]	0.94 [0.61, 1.45]
High school/2 years college	1.25 [0.92,1.70]	0.93 [0.65, 1.33]	0.82 [0.52, 1.27]
Unknown	1.52 [0.80, 2.90]	0.75 [0.32, 1.77]	0.85 [0.24, 3.00]

Sex^a^			
Female	1.00 [0.81, 1.22]	1.06 [0.83, 1.35]	1.21 [0.88, 1.64]
Unknown	1.12 [0.49, 2.59]	3.30 [1.08, 10.15]^*^	1.19 [0.16, 8.70]

Spoken English ability			
Low	1.76 [1.36, 2.27]^***^	1.24 [0.90, 1.69]	0.73 [0.45, 1.20]
Unknown	1.51 [0.70, 3.25]	2.22 [0.89, 5.54]	3.10 [1.07, 9.00]^*^

Age (years)			
45–64	1.29 [1.07, 1.55]^**^	0.77 [0.60, 0.98]^*^	1.26 [0.92, 1.71]
65+	1.12 [0.67, 1.88]	0.62 [0.32, 1.20]	1.13 [0.47, 2.69]
Unknown	3.67 [1.82, 7.40]^***^	0.36 [0.12, 1.09]	0.54 [0.08, 3.75]

Smoking status			
Yes	1.38 [1.12, 1.71]^**^	1.06 [0.82, 1.37]	1.26 [0.89, 1.77]
Unknown	1.14 [0.65, 1.99]	0.78 [0.33, 1.84]	1.37 [0.49, 3.82]

Chronic health condition	3.05 [2.47, 3.78]^***^	0.86 [0.68, 1.10]	1.24 [0.87, 1.77]

Mental health condition	2.23 [1.83, 2.70]^***^	1.33 [1.02, 1.74]^*^	1.11 [0.83, 1.50]

Other health condition	3.30 [2.77, 3.95]^***^	0.83 [0.66, 1.05]	1.69 [1.23, 2.32]^**^

Race and ethnicity			
Latino/a/e and Hispanic	1.46 [1.14, 1.87]^**^	1.44 [1.02, 2.03]^*^	1.05 [0.67, 1.63]
African American/Black	1.29 [0.98, 1.70]	1.30 [0.88, 1.91]	1.23 [0.76, 1.97]
Asian, Native Hawaiian/Pacific	0.85 [0.65, 1.12]	1.65 [1.20, 2.27]^**^	2.19 [1.49, 3.23]^***^
Islander, and American Indian/Alaska Native			
Race and ethnicity not reported	0.82 [0.39, 1.72]	1.03 [0.35, 3.01]	1.10 [0.34, 3.57]

New York City indicator	0.92 [0.77, 1.11]	1.24 [0.96, 1.59]	0.96 [0.68, 1.36]

Note. OHL = organizational health literacy.

a Reference groups include: male, White, 4+ years college, high spoken English ability, and age 18–44 years.

* p < .05.

** p < .01.

*** p < .001.

Three MMC plans out of 16 had statistically significantly different average health literacy rates compared to the statewide average. Two plans had lower OHL rates while one plan had a higher OHL rate. Qualitatively, the significantly higher HL group of plans tended to consist of younger members, more educated members, more White members, and more members who spoke English better compared to the significantly lower health literacy group (See **Table 4**).

**Table 4:**
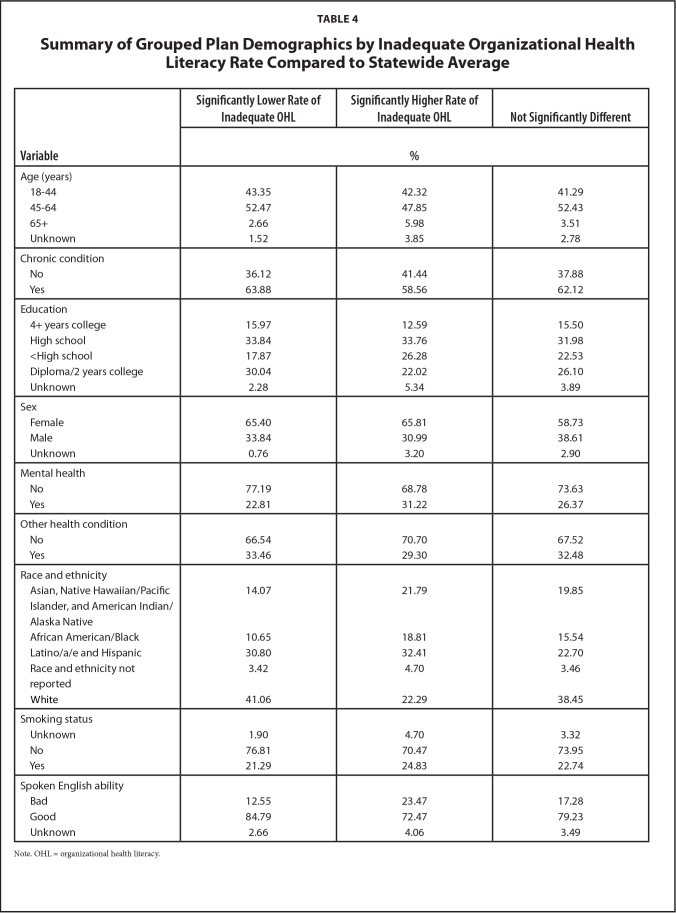
Summary of Grouped Plan Demographics by Inadequate Organizational Health Literacy Rate Compared to Statewide Average

**Variable**	**Significantly Lower Rate of Inadequate OHL**	**Significantly Higher Rate of Inadequate OHL**	**Not Significantly Different**

	**%**		

Age (years)			
18–44	43.35	42.32	41.29
45–64	52.47	47.85	52.43
65+	2.66	5.98	3.51
Unknown	1.52	3.85	2.78

Chronic condition			
No	36.12	41.44	37.88
Yes	63.88	58.56	62.12

Education			
4+ years college	15.97	12.59	15.50
High school	33.84	33.76	31.98
<High school	17.87	26.28	22.53
Diploma/2 years college	30.04	22.02	26.10
Unknown	2.28	5.34	3.89

Sex			
Female	65.40	65.81	58.73
Male	33.84	30.99	38.61
Unknown	0.76	3.20	2.90

Mental health			
No	77.19	68.78	73.63
Yes	22.81	31.22	26.37

Other health condition			
No	66.54	70.70	67.52
Yes	33.46	29.30	32.48

Race and ethnicity			
Asian, Native Hawaiian/Pacific	14.07	21.79	19.85
Islander, and American Indian/Alaska Native			
African American/Black	10.65	18.81	15.54
Latino/a/e and Hispanic	30.80	32.41	22.70
Race and ethnicity not reported	3.42	4.70	3.46
White	41.06	22.29	38.45

Smoking status			
Unknown	1.90	4.70	3.32
No	76.81	70.47	73.95
Yes	21.29	24.83	22.74

Spoken English ability			
Bad	12.55	23.47	17.28
Good	84.79	72.47	79.23
Unknown	2.66	4.06	3.49

Note. OHL = organizational health literacy.

## Discussion

The current study estimated that 20% of participants experienced inadequate OHL. Although this is not the majority of clients, it means that 1 of 5 people are facing communication barriers during health care encounters. Of greater concern is that this 20% is more likely to include members who already have risk factors for health disparities, such as Latino/a/e and Hispanic populations, those with lower education levels, and members with mental health conditions.

Our results indicated differences in demographic distributions between plans that differed from the statewide inadequate OHL rate. Qualitatively these demographic differences included the variables: race and ethnicity (Chaudhry et al., 2011; Kutner et al., 2006), education level, and English language ability (Kutner et al., 2006). This study suggests that it is possible to leverage existing data that is collected annually across the U.S. to gain a greater understanding of organizational health literacy.

Although at first glance the sex imbalance could have an impact on the validity of the results, the ratio accurately represents the Medicaid population in New York. Plans are sampled proportionally for sex and the response rates are similar for each sex. In addition, several studies have found that CAHPS questionnaire does not have differential item functioning by sex (Elliott et al., 2012; Rodriguez & Crane, 2011; Setodji et al., 2019).

We found that lower OHL is linked with lower perceived access to health care. This matches with prior research of individual health literacy for Medicare members (Levy & Janke, 2016; Morgan et al., 2008). The strong association (*OR* = 6.97; Haddock et al., 1998) in this study indicated the Medicaid population may perceive a large barrier to health care access, even though comprehensive coverage is provided by NYS Medicaid program. A lack of perceived access may cause members to not seek care, delay care, or use unnecessary services which can lead to worse health conditions, require costly inpatient services, and financially burden the Medicaid program.

Additionally, Medicaid members reporting inadequate OHL were more likely to report less satisfaction with their overall health care and poorer health even after statistically controlling for the presence of health conditions (chronic, mental, and other), as well as demographics. While these findings are supported by prior research looking at individual health literacy (Altin & Stock, 2015; Furuya et al., 2015; Ownby et al., 2012; Shea et al., 2006), we observed stronger risks for the Medicaid population when focusing on OHL. Health care organizations should develop a focused strategy for improving OHL especially for organizations serving many Medicaid patients.

It is important to consider that health literacy is not only determined by an individual's abilities, but also by the health care environment they navigate (Brach, 2017; Brach et al., 2012; Parker & Ratzan, 2010). This was formalized with the definition of health literacy in Healthy People 2030 (Brach & Harris, 2021; Office of Disease Prevention and Health Promotion, n.d.). Unclear instructions from providers could lead to misunderstandings about needed care and how to access care, which can result in low satisfaction and worse health. Ensuring providers use strategies such as Teach Back (Yen & Leasure, 2019) and plain language may enhance members' satisfaction and health outcomes. Helping health care organizations become health literate can lead to improved patient experience, and thus patient satisfaction. It can also benefit the plans themselves, as plans with superior performance receive an incentive to their per member per month payments (New York State Department of Health, 2018b).

Research (Brach et al., 2012) has sought to identify ways in which health care organizations can become more health literate. Organizations can use the variety of available tools to assess the health literacy of their organization and identify areas for improvement. This requires commitment from the leadership of an organization and incorporation principles of health literacy into the “organizations' mission, vision, and strategic planning” (Farmanova et al., 2018). In one scoping review, “leadership support, top-down and bottom-up approaches, a change champion, and staff commitment” were necessary elements to ensure change (Kaper et al., 2021). Written communication can be improved by providing organizations with inadequate OHL with the toolkit for making written material clear and effective from the Centers for Medicare & Medicaid Services (n.d.).

## Study Limitations

The main limitation of the study was that, although the two questions used as a screening measure captured the challenges in the areas of communication and understanding information, they do not reflect all aspects of OHL. Without significantly increasing the burden on members, some other survey items in the CAHPS Health Literacy Item Set can be considered for measuring the organization's progress in this area (e.g., the question for help with forms used in the Agency for Healthcare Research and Quality's Medical Expenditure Panel Survey) (Liang & Brach, 2017).

In addition, the unique characteristics of the NYS Medicaid population may not represent other populations. Generalization of the measure and findings need to be further tested. The self-reported outcomes may also limit our conclusions on health and access to care, as they are not based on objective medical or appointment records. However, many of the existing OHL measures are based on self-report and have been validated.

Also, the reason respondents who had valid skips for both questions is unknown. It is plausible that some respondents had valid skips due to an issue with access to care. Incorporating multiple imputation allowed us to include all the participants who responded to the survey, instead of removing them for having an incomplete survey.

## Future Research

Future research should collect more validity evidence to support the use of a brief set of CAHPS questions as a measure of OHL. Validity evidence already exists for both items, however, using just two items from CAHPS has not been done previously. Ideally, future research on convergent validity would show a relationship between these questions and other measures of OHL, and discriminant validity would provide evidence that the questions are not correlated with conceptually unrelated constructs.

Research could also explore the use of these questions in other states to see if the results are generalizable, as it would provide an inexpensive and noninvasive way to assess OHL for service sites used by the Medicaid population. Utilizing the current protocol would only require that states add one question to the survey, as the primary doctor question is a core question within the regular CAHPS survey. Allowing OHL data collection to be part of existing data efforts would help organizations and systems with identifying where interventions and resources should be focused (U.S. Department of Health and Human Services, Office of Disease Prevention and Health Promotion, 2010) to improve patients' experience with the health care system, as well as patients' health outcomes.
